# IgA vasculitis with nephritis in cirrhotic Wilson disease: Is there an association? 

**DOI:** 10.5414/CNCS110268

**Published:** 2020-10-12

**Authors:** Ratna Acharya, Xu Zeng, William L. Clapp, Kiran Upadhyay

**Affiliations:** 1 Division of Pediatrics,; 2 Division of Anatomic Pathology, Department of Pathology, and; 3 Division of Pediatric Nephrology, Department of Pediatrics, University of Florida, Gainesville, FL, USA

**Keywords:** IgA vasculitis, nephritis, Wilson disease, child

## Abstract

Introduction: IgA vasculitis (IgA-V) predominantly involves skin, gastrointestinal (GI) tract, joints, and kidneys. Wilson disease (WD) is a hepatolenticular degenerative disease caused by *ATP7B* gene mutation. Case report: Here we describe an unusual association of IgA-V with nephritis (IgA-VN) in an 11-year-old child with WD. He presented with palpable purpura without arthritis and GI involvement. Renal function was normal. Urinalysis showed microscopic hematuria and tubular proteinuria. Evaluation showed transaminitis, hypoalbuminemia, IgA hyperglobulinemia, and coagulation abnormalities. Serum ceruloplasmin and copper were low and 24-hour urine copper was extremely elevated. Liver biopsy showed stage IV cirrhosis with increased quantitative liver copper content. Skin and renal biopsy showed IgA-positive leukocytoclastic vasculitis and mesangial hyperplasia with IgA deposition, respectively. Quantitative renal copper content was normal. Homozygous pathogenic variant c.3207C>A (p.His1069Gln) of *ATP7B* was detected. There were no Kayser-Fleischer rings in the eyes, and neuropsychiatric examination was normal. Treatment with zinc and trientine led to normalization of hepatic function and serum IgA level with resolution of the rash and maintenance of renal function. Conclusion: Defective hepatic processing and/or clearance of IgA/IgA immune complexes probably led to the IgA-mediated skin and renal injury. Further such reports will help augment our understanding on the pathophysiology of IgA-VN in WD.

## Introduction 

IgA vasculitis (IgA-V), also called Henoch-Schonlein purpura, is the most common small vessel vasculitis in children [1]. Mesangial IgA deposits are the hallmark renal lesion of IgA-V with nephritis (IgA-VN), as in IgA nephropathy (IgA-N). Wilson disease (WD) is a rare, autosomal recessive copper metabolism disorder with excess hepatic and extra-hepatic copper accumulation [2]. Renal involvement can occur in up to 5% of children with WD [3]. Tubular dysfunction is the most common renal manifestation; refractory rickets and nephrocalcinosis can also occur [4, 5]. A few pediatric cases of IgA-N without IgA-V associated with WD have been reported [6, 7]. Similarly, a report of a child with IgA-VN and WD with both renal failure and end-stage cirrhosis has been published [8]. In adults, several cases of IgA-VN in association with hepatitis C induced cirrhosis have been described [9, 10, 11]. Here we report a case of mild IgA-VN in a child with cirrhotic WD who had reversal of hepatic function with copper chelation. 

## Case presentation 

A previously healthy 11-year-old Hispanic boy presented with a history of diffuse skin rash in the lower extremities which started 3 months ago. The non-pruritic and non-painful rash resolved a week after the onset but reappeared a month ago, evolving from his ankles and progressing up to the gluteal region and arms. There was no history of fever, cough, sore throat, abdominal or joint pain, edema, or gross hematuria. There was no prior history of epistaxis, hematemesis, bloody or pale stool, pruritus, jaundice, fatigue, appetite changes, prolonged bleeding after injury, or easy bruising. Family history was significant for psoriasis in the father and celiac disease in the paternal grandmother. His primary physician had started him on oral prednisone 60 mg daily a month ago without improvement in the rash. During the visit to a pediatric nephrologist office, the initial vital signs were normal with a blood pressure (BP) of 104/62 mmHg (< 95^th^ centile for his age, gender, and height). Physical examination was normal except for a non-blanching palpable purpura in the gluteal region, arms and legs, sparing the trunk, face, palms, and soles. Urinalysis showed pH of 5.5, specific gravity 1.015, glycosuria, 2+ proteinuria with a protein-to-creatinine ratio of 0.5, and microscopic hematuria (70 red blood cells (RBC) per high power field (HPF)) with RBC casts. Urine microalbumin-to-creatinine ratio was 16 mg/g creatinine, and urine β2 microglobulin was 15,150 µg/L (normal 0 – 300 µg/L). Urine calcium-to-creatinine ratio was 0.18 and the tubular reabsorption of phosphate was 90%. Renal function test showed blood urea nitrogen 9 mg/dL, serum creatinine 0.5 mg/dL, and normal electrolytes. Blood counts were normal. Liver function test showed the following: alanine aminotransferase (ALT) 78 U/L (normal: 8 – 30 U/L), aspartate aminotransferase (AST) 113 U/L (normal: 12 – 32 U/L), albumin 2.4 gm/dL, total bilirubin 0.8 mg/dL, alkaline phosphatase 64 U/L (normal: 91 – 476 U/L), and γ glutamyltransferase levels 144 U/L (normal: 3 – 22 U/L). Serum ammonia and vitamin K were normal. Prothrombin time (PT) and partial thromboplastin time (PTT) were 24.1 seconds (international normalized ratio, INR 2.1) and 52 seconds, respectively; both normalized with mixing study. Plasma clotting factor activities were all low except fibrinogen and factor VIII activity. Von Willebrand factor (VWF) antigen and activity were normal along with a normal distribution of VWF multimer. Lupus anticoagulant and anticardiolipin antibodies were negative. Serum haptoglobin was undetected (< 30 mg/dL). Direct coombs test was negative. 

Further investigations showed normal serum liver-kidney microsomal and anti-smooth muscle IgG antibody, tissue transglutaminase IgA antibody, and α-1 antitrypsin level. Hepatitis A IgM, hepatitis B surface antigen, hepatitis B core IgM and hepatitis C antibodies, and cryoglobulins were negative. Serum ceruloplasmin level was 14 mg/dL (normal 18 – 35 mg/dL), and serum copper was 18 µg/dL (normal: 72 – 166 µg/dL). 24-hour urine copper level was 1,285 µg/g creatinine (normal: 10 – 45 µg/g creatinine). All infectious etiologies were negative. Serum C3 and C4 levels were 60 mg/dL (normal: 90 – 200 mg/dL) and 9 mg/dL (normal: 13 – 50 mg/dL), respectively. Antinuclear, anti-double stranded DNA, rheumatoid factor, and anti-nuclear cytoplasmic antibodies were negative. 

Liver sonogram and magnetic resonance imaging (MRI) showed a micronodular echotexture with a good hepatopetal flow in the main portal vein. There was evidence of mild to moderate hepatic fibrosis in the shear wave elastography (SWE), with a METAVIR score of F2. Core needle liver biopsy showed evidence of stage IV cirrhosis (Scheuer classification) and a faint focal hepatocellular copper staining. Quantitative liver copper quantification was 600.8 µg/g dry weight of liver tissue (normal: 15.5 – 55 µg/g). Evaluation of *ATP7B* gene for sequence changes and exonic deletion/duplication showed a homozygous pathogenic variant c.3207C>A (p.His1069Gln) (Invitae Wilson’s disease testing, San Francisco, CA, USA). Based upon the biochemical findings and genetic testing, the diagnosis of WD was confirmed. A slit-lamp eye examination did not show Kayser-Fleischer rings. He was started on trientine hydrochloride 500 mg twice daily and zinc gluconate 200 mg twice daily along with copper-free diet. Prednisone was discontinued after a tapering course. 

**Figure 1.**
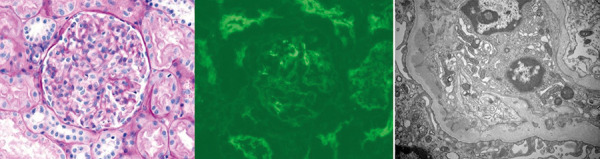
Renal histology showing segmental minimal mesangial hypercellularity (Periodic acid-Schiff stain, 400×), mesangial IgA deposition (immunofluorescence-IgA, 400×), and paramesangial electron-dense deposits (electron microscopy, 15,000×).

A punch skin biopsy from the purpuric lesions showed leukocytoclastic vasculitis along with granular deposits of IgA and C3 in the walls of small vessels. Renal bladder sonogram showed right kidney of 10.3 cm and left kidney of 10 cm in length with no calculus, nephrocalcinosis, hydronephrosis, cyst, or mass. Percutaneous renal biopsy showed focal segmental mild mesangial hypercellularity, mild tubular atrophy and interstitial fibrosis (10%), minimal interstitial inflammation, and tubular RBC casts on light microscopy. Immunofluorescence showed segmental granular mesangial staining for IgA (3+), C3 (3+), and IgG (1+). Electron microscopy confirmed the dense paramesangial deposits with intact podocyte foot processes (Figure 1). A diagnosis of IgA-N was made with an Oxford classification of M0 E0 S0 T0 C0. Renal copper quantification was normal (10.6 µg/g dry weight). Serum immunoglobulins showed IgA 488 mg/dL (64 – 246 mg/dL), IgG 2,423 mg/dL (normal: 820 – 1,835 mg/dL), and normal IgE and IgM levels. 

**Figure 2.**
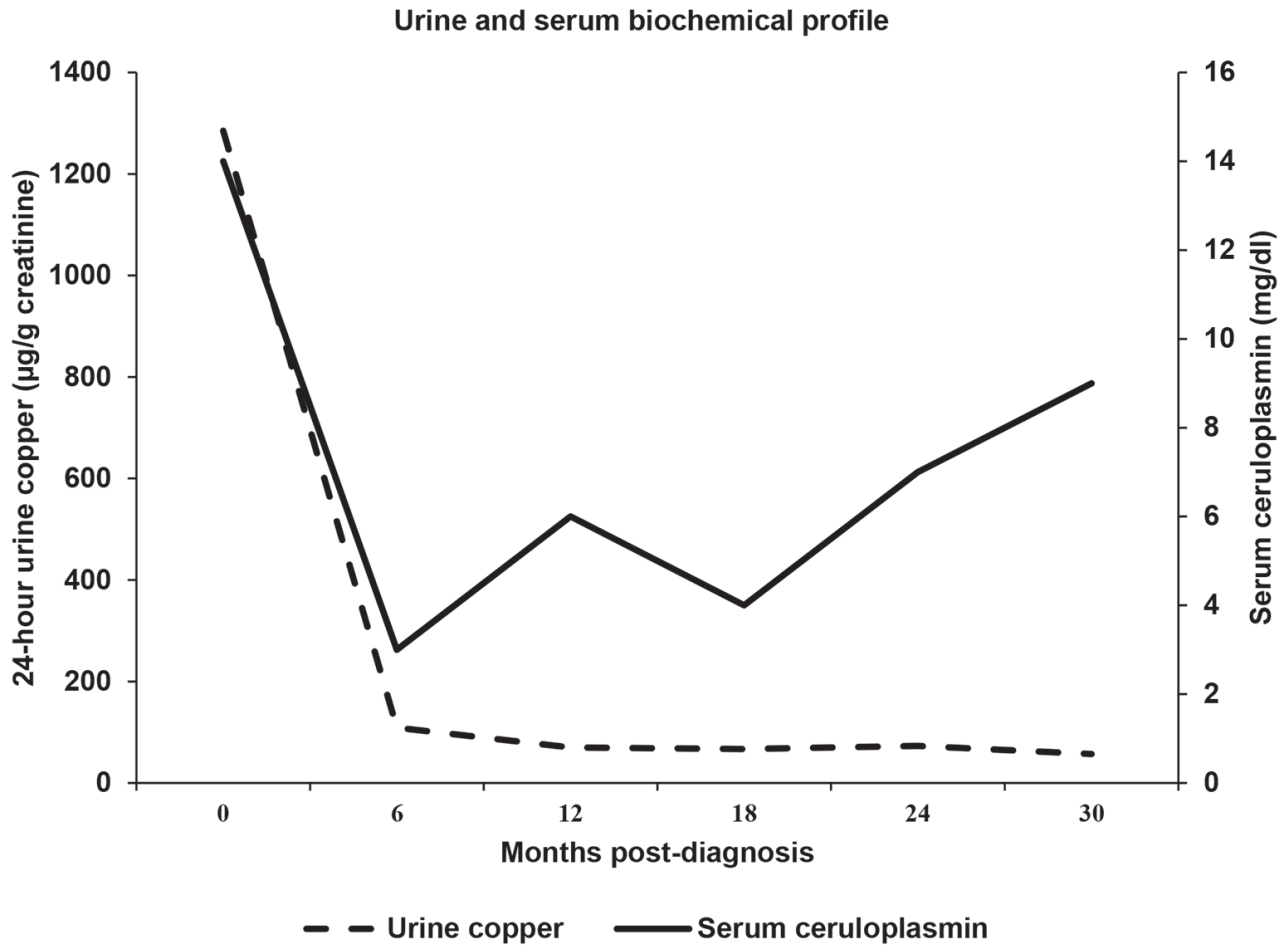
Marked reduction in urine copper and stabilization of serum ceruloplasmin with copper chelation.

During a 30-month follow-up period, his palpable purpura resolved and liver enzymes normalized between 3 and 6 months after initiation of trientine and zinc. 24-hour urine copper remained markedly suppressed (Figure 2). Although the serum ceruloplasmin was persistently low, the non-ceruloplasmin-bound copper (free copper) remained at goal (most recently, 5.6 µg/dL; goal: 5 – 15 µg/dL). Follow-up liver sonogram and SWE after 2 years showed a homogenous echotexture of liver without evidence of cirrhosis and a METAVIR fibrosis score of F0. Urinalyses continued to show absence of albuminuria and glycosuria, resolution of tubular proteinuria, and decreasing microscopic hematuria (most recently 30 RBC per HPF). Renal function and BPs remained stable throughout. Serum IgA and IgG normalized. Apart from a diagnosis of generalized anxiety disorder and mild depression 18 months later, his neuropsychiatric status remained stable. Brain MRI and electroencephalogram were normal. He remains on trientine hydrochloride 500 mg twice daily. 

## Discussion 

Palpable purpura in the absence of coagulopathy or thrombocytopenia is the mandatory criterion for diagnosis of IgA-V along with presence of one or more of the following: acute-onset diffuse abdominal pain, acute arthritis/arthralgia, renal involvement, and IgA-dominant leukocytoclastic vasculitis or proliferative glomerulonephritis [12]. Hepatic dysfunction usually does not occur secondary to IgA-V. Hence, the presence of coagulopathy and transaminitis in a child with IgA-V should raise the possibility of associated primary liver disease, as in our case, and needs further investigation. 

Secondary IgA nephropathy (sIgA-N) is known to occur in association with alcoholic cirrhosis (also called cirrhotic glomerulonephritis) in adults [13]. Increased IgA synthesis along with defective hepatic processing and/or clearance has been described in these patients [14, 15]. As in primary IgA-N, these abnormally glycosylated IgA1 form large soluble IgA1 immune complexes (IC) by combining with IgG and IgA autoantibodies, which then deposit in the renal mesangium leading to mesangial injury. Whether the similar pathophysiology of renal injury can be extrapolated in children with WD needs to be studied further. However, given similar liver histology in our patient as in alcoholic cirrhosis, it is reasonable to hypothesize that the increased IgA synthesis along with decreased IgA-IC clearance by hepatic mononuclear phagocytes could play a role in inducing sIgA-N. In IgA-VN, in addition to the renal mesangium, these IgA-ICs also get deposited in the small vessels of the skin, joints, and GI tract, activating neutrophils via the IgA Fc receptor FcαRI (CD89), and ultimately causing tissue damage, resulting in palpable purpura, arthritis, and GI hemorrhage [16]. Indeed, 37% of children with IgA-VN, including our case, have elevated serum IgA level [16]. Hence, it is possible that once serum IgA normalizes with stabilization of hepatic function after adequate copper chelation, the purpura also resolves, as seen in our patient. However, this needs to be studied further in future studies. 

Reports on direct renal effects of copper are limited to few case studies. Chugh et al. [17], in their case series described 11 out of 29 patients who developed acute kidney injury from copper sulfate intoxication; 10 required dialysis. Renal histology showed acute tubular necrosis. Our patient had chronic copper overload from WD but had normal renal function, and the renal biopsy revealed IgA mesangial deposits with mild tubular epithelial cell degeneration and atrophy. The tubular proteinuria most likely was secondary to excess copper-related mild renal tubular epithelial cell dysfunction and resolved after treatment with copper-chelating agent [18]. There was no glomerular proteinuria. Wolff et al. [19] studied renal histological changes in postmortem specimens of 5 WD patients who showed normal glomeruli but degeneration of tubular epithelial cells. Copper deposition was not uniform throughout the kidney but wherever present, rubeanic acid staining showed intracytoplasmic copper granules in the tubular epithelial cells [19]. Although renal copper staining was not done, the renal biopsy copper quantification was not elevated in our patient. 

Copper chelating agents with or without zinc supplementation are the mainstay of therapy in WD [20]. Whether these therapies are also efficacious in reversing nephritis in those with associated IgA-N is not very clear, although some studies have shown clear benefits [6, 21]. Our patient remained without gross hematuria and proteinuria, and with normal renal function and BP during the 30-month follow-up. Zinc and trientine, by stabilizing the hepatic function and thereby reducing the circulating IgA-ICs, could have caused the resolution of skin IgA vasculitis and preservation of renal function in our patient. 

## Funding 

This study received no funding. 

## Conflict of interest 

The authors declare no conflict of interest.
